# Factors associated with loss of vertebral height and kyphosis correction after intermediate screws in short segment pedicular fixation for type-A fractures of the thoracolumbar spine: A retrospective study

**DOI:** 10.1097/MD.0000000000038343

**Published:** 2024-05-31

**Authors:** Junchao Zhang, Zhou Ye, Yi Mao

**Affiliations:** a Department of Orthopaedics, The Quzhou Affiliated Hospital of Wenzhou Medical University, Quzhou People’s Hospital, Quzhou, Zhejiang Province, China.

**Keywords:** correlation, kyphosis, spine, thoracolumbar fracture, vertebral height

## Abstract

In this article, we attempted to identify risk factors affecting the loss of vertebral height and kyphosis correction on type A thoracolumbar fractures. Patients with type A thoracolumbar fractures who underwent short segments with intermediate screws at the fracture level management between 2017 and 2022 were included in this study. Clinical factors including patients’ demographic characteristics (age, sex), history (smoking, hypertension and/or diabetes), value of height/kyphosis correction, the thoracolumbar injury classification and severity score (TLICS), the load sharing classification (LSC) scores and bone mineral density were collected. Correlation coefficient, simple linear regression analysis and multivariate regression analysis were performed to identify the clinical factors associated with the loss of vertebral height/kyphosis correction. Finally, 166 patients were included in this study. The mean height and kyphosis correction were 21.8% ± 7.5% and 9.9° ± 3.8°, respectively, the values of the loss were 6.5% ± 4.0% and 3.9° ± 1.9°, respectively. Simple linear regression analysis and multivariate regression analysis showed that age, value of height correction, LSC scores and bone mineral density were significantly associated with the loss of vertebral height and kyphosis correction (*P* < .01) We could draw the conclusion that patients with older age, lower bone mineral density, higher LSC scores and diabetes are at higher risk of vertebral height and kyphosis correction loss increase. For these patients, appropriate clinical measures such as long segment fixation, control of blood glucose, and increase of bone density must be taken to reduce the loss of correction.

## 1. Introduction

Thoracolumbar fractures are the most common fractures in the spine, accounting for nearly 90%.^[1,2]^ However, the management of thoracolumbar injuries remains controversial to date.

Surgical management has the advantage of restoring spine stability, better-correcting deformities, and early mobilization.^[3,4]^ The surgical method remains a matter of debate, and the main bone of contention is short or long-segment posterior fixation.^[5]^

Posterior short segment fixation (1 level above and 1 level below the fracture level) is considered less invasive and to prevent adjacent discs from overloading, but it also couples with an unacceptably high rate of internal fixation failure and kyphotic collapse.^[6,7]^ Posterior long segment fixation (2 levels above and 2 levels below the fracture level) enhances the stability and could achieve effective correction of kyphosis, at the expense of prolonging the operative time, increasing the amount of bleeding, higher cost and the load on adjacent discs.^[5,8]^

Short segments with intermediate screws at the fracture level have been proven to increase structural stiffness and protect fractured vertebrae from anterior loading.^[9]^ However, a significant loss of kyphosis and height correction were found clinically. In this paper, we sought to identify the factors associated with the loss of vertebral height and kyphotic correction of type A thoracolumbar fractures in order to identify high-risk patients and reduce the incidence of correction loss through early intervention.

## 2. Methods

### 2.1. Study design

Patients with type A thoracolumbar fractures who underwent short segments with intermediate screws at the fracture level management from January 1, 2017 to October 1, 2022 were enrolled. This study was approved by the Medical Ethics Committee of Quzhou People Hospital (Ethics approval number: 2021-185) prior to patient enrollment. All patients were informed about the purpose and procedures of the study and provided informed consent.

### 2.2. Inclusion criteria

The inclusion criteria were as follows: patients aged from 20 to 75 years; patients with single-level type A (according to the new AO spine classification^[10]^) thoracolumbar fractures; patients treated with short segment fixation with intermediate screws at the fracture level; follow-up ≥ 6 months.

Exclusion criteria included fracture type other than A, multilevel fractures, pathological fracture (infection, tumor metastasis and so on), anterior or long-segment posterior fixation and follow-up < 6 months.

### 2.3. Operative intervention

All patients underwent surgery within 3 days of admission. Under general anesthesia, patients were positioned prone in the hyperlordotic position. The fracture vertebral level was identified by X-ray fluoroscopy. A Wiltse approach was performed.^[11]^ Pedicle screws were placed a level above and below the fracture, and then 2 intermediate screws were placed into the fracture level. The length and diameter of pedicle screws were selected according to the characteristics of different fixation segments and preoperative computerized tomography measurements. Fracture reduction and vertebral height correction were achieved with rod over contouring or distraction, followed by tightening of the construct. In fractures with neurologic deficits or spinal canal compromise >50%, a standard posterior midline approach and a decompressive laminectomy were performed.

### 2.4. After surgery

Stitches are removed about 10 to 14 days after surgery. All patients were mobilized with brace protection the day after surgery for 3 months. All patients were followed up 1, 3, and 6 months after surgery. Anterior-lateral radiographs centered on the fracture segment were performed at the last follow-up.

### 2.5. Clinical data collection

**Figure 1.**
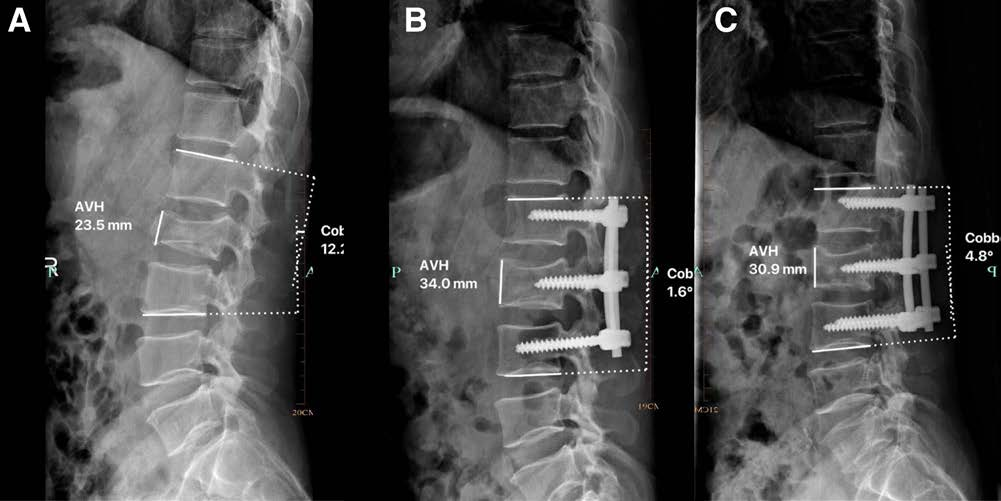
Pre-operative (A), post-operative (B) and follow-up (C) radiological measurements on a lateral radiograph, including anterior body height and Cobb angle.

Clinical data including patients’ demographic characteristics (age, sex), history (smoking, hypertension and/or diabetes), preoperative bone mineral density (BMD), level of fractures, the type of trauma, neurological status, and the preoperative, postoperative and 6-month follow-up radiographs, were recorded. Areal BMD at the femoral neck, total hip, and lumbar spine (L2-L4) was measured by a dual-energy X-ray absorptiometry. Each fracture was independently classified by 2 blinded authors, including The Thoracolumbar Injury Classification and Severity Score (TLICS)^[12]^ and the load sharing classification (LSC).^[13]^ The American Spinal Injury Association (ASIA) classification system^[14]^ was used to determine the neurological status of the patients. A preoperative computed tomography scan was performed to assess the fracture pattern. Plain radiograph analysis included measurements of anterior body height and local kyphosis. The percentage of anterior body height compression was calculated using the methods introduced by Mumford et al.^[15]^ Local kyphosis was measured between the superior endplate of the upper and the inferior endplate of the lower adjacent vertebrae by the Cobb method (Fig. 1). Functional outcome was assessed by Oswestry disability index (ODI) questionnaire. Back pain was quantified using a visual analogue scale (VAS).

### 2.6. Statistical analysis

SPSS 26.0 statistical software (IBM, Chicago, IL) was used for data analysis. Pearson correlation coefficient was used to analyze the correlation with the loss of vertebral height/kyphosis correction and the variables. Logistic regression was also used to analyze any significant associations (*P* < .05). Unpaired Student *t* test was used to compare the means of normally distributed data.

## 3. Results

One hundred and sixty-six patients with type A thoracolumbar fractures who underwent short segments with intermediate screws at the fracture level management were included. Out of the 166 cases, there were 116 males (69.9%) and 50 females (30.1%). The mean age was 51.9 ± 10.2 years, respectively. The affected levels were T11 (n = 20, 12.0%), T12 (n = 48, 28.9%), L1 (n = 74, 44.6%), and L2 (n = 24, 14.5%). The fracture types were A1 (n = 14, 8.4%), A2 (n = 13, 7.8%), A3 (n = 91, 54.8%), and A4 (n = 48, 28.9%). 32 patients accompanied with neurological deficits (6 in ASIA C, 26 in ASIA D). The mean height and kyphosis correction were 21.8% ± 7.5% and 9.9° ± 3.8°, respectively, the values of the loss were 6.5% ± 4.0% and 3.9° ± 1.9°, respectively. Other demographic information is presented in Table 1.

**Table 1:** The demographic characteristics of included patients.

Characteristic	n (%)	*P* value	
		LVHC	LKC
Sex		0.457	0.336
Male	103 (62.0)		
Female	63 (38.0)		
Diabetes		0.048	0.019
Yes	38 (22.9)		
No	128 (77.1)		
Bone mineral density		<0.001	<0.001
>−2.5	21 (12.7)		
≤−2.5	145 (87.3)		
Hypertension		0.256	0.197
Yes	43 (25.9)		
No	123 (74.1)		
Smoking history		0.191	0.239
Yes	53 (31.9)		
No	113 (68.1)		
Decompressive laminectomy		0.017	<0.001
Yes	32 (31.3)		
No	134 (68.7)		
Body mass index		0.076	0.112
<25	127 (76.5)		
≥25	39 (23.5)		

LKC = loss of kyphosis correction, LVHC = loss of vertebral height correction.

### 3.1. Clinical outcomes

VAS scores were 6.4 ± 1.4 pre-operatively, and significantly decreased to 2.1 ± 0.9 during the post-operative period. At the follow-up periods that is 0.7 ± 0.2.

Before and after operation and during follow-up, the ODI scores were 77.4 ± 7.6, 25.8 ± 6.6, and 9.9 ± 3.2.

### 3.2. Factors affecting the loss of vertebral height correction

**Figure 2.**
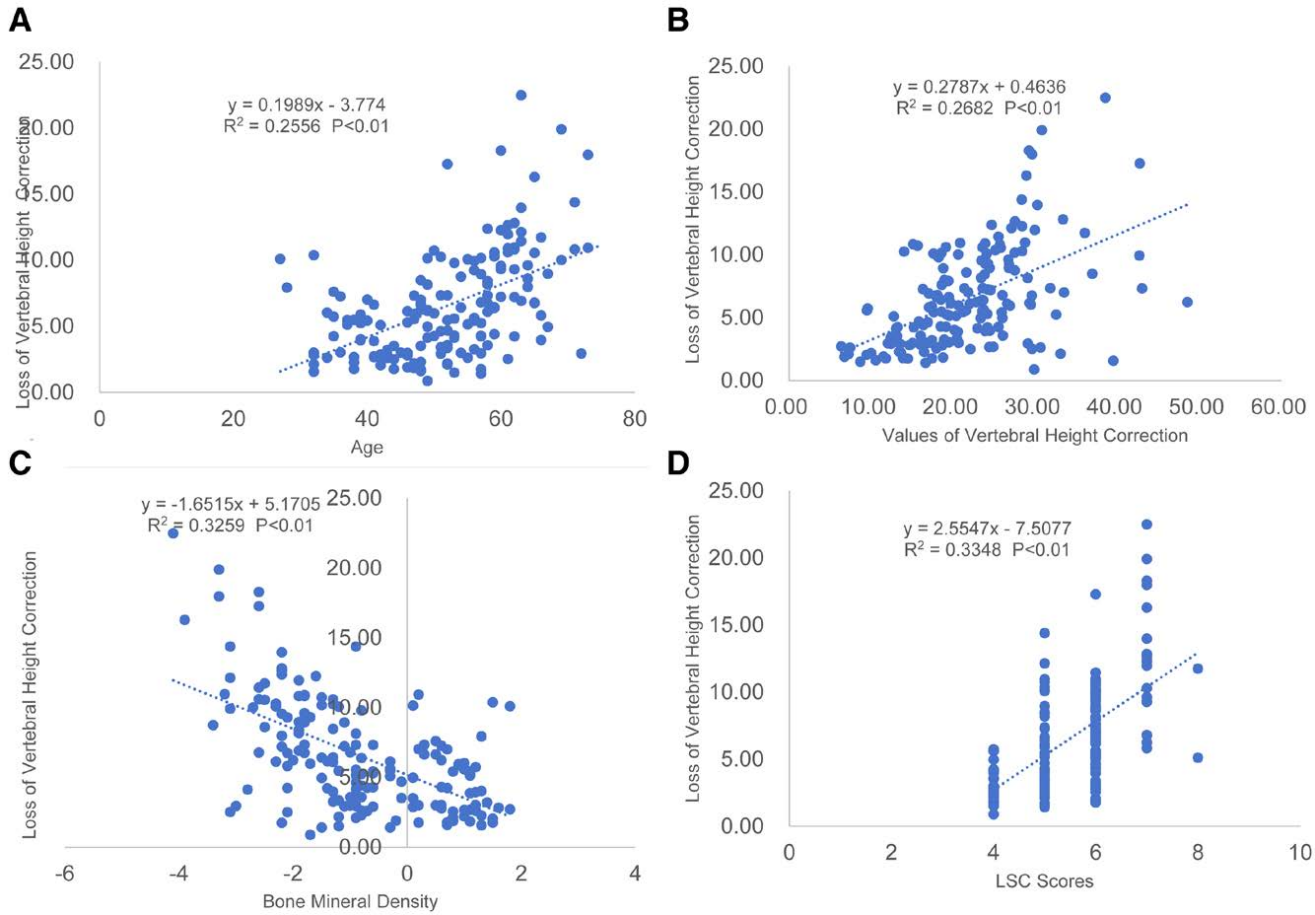
Scatter plots and linear regression values for the significant correlations with the loss of vertebral height correction. (A) A significant correlation is shown between the loss of vertebral height correction and the age of patients. (B) A significant correlation is shown between the loss of vertebral height correction and the values of kyphosis correction. (C) A significant correlation is shown between the loss of vertebral height correction and bone mineral density. (D) A significant correlation is shown between the loss of vertebral height correction and the LSC scores. LSC = the load sharing classification.

**Figure 3.**
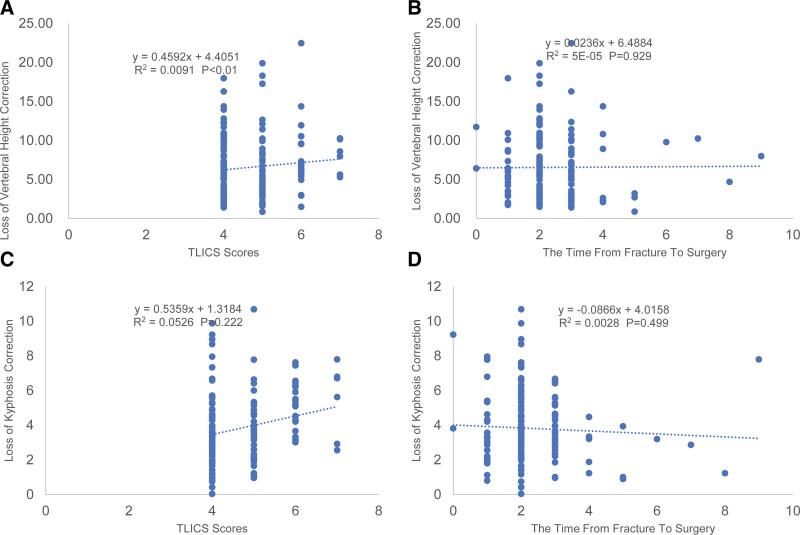
Scatter plots and linear regression values for the significant correlations with the loss of vertebral height/kyphosis correction. (A) A significant correlation is shown between the loss of vertebral height correction and the TLICS scores. (B) No significant correlation is observed between the loss of vertebral height correction and the time from fracture to surgery. (C) No significant correlation is observed between the loss of kyphosis correction and the TLICS scores. (D) No significant correlation was observed between the loss of kyphosis correction and the time from fracture to surgery. TLICS = the thoracolumbar injury classification and severity score.

There was no significant sex difference in the loss of vertebral height correction (*P* > .05). Diabetes and decompressive laminectomy were risk factors in the loss of vertebral height correction (*P* < .05). Age, values of height correction, TLICS and LSC scores were significantly and positively associated with loss of vertebral height correction, which was described by linear regression analysis to have a predictive value of 50.6% (Fig. 2A), 51.8% (Fig. 2B), 9.5% (Fig. 3A), and 57.9% (Fig. 2D), respectively. There was a significant and negative correlation between BMD and loss of vertebral height correction, with a 57.6% (Fig. 2C) predictive value. No significant correlation was observed between the loss of vertebral height correction and the time from fracture to surgery (Fig. 3B). Simple linear regression analysis showed that age, value of height correction, TLICS scores, LSC scores and BMD were all the significant predictors of the loss of vertebral height correction.

Significant relationships (*P* < .05) were subjected to multivariate regression analysis. Considering that there was a significant correlation between age and BMD, they were assigned to 2 statistical models. The results suggested that age, BMD, value of height correction and LSC scores were constant significant predictors of the loss of vertebral height correction, but TLICS scores were no longer the predictors (Table 2).

**Table 2:** Multivariable linear regression to factors affecting the loss of vertebral height correction.

Parameters	UC		SC	t	Sig.	95% CL for Beta		Adjusted *R*^2^
	Beta	Std. Error	Beta			Lower bound	Upper bound	
Model 1								0.510
HC	0.161	0.032	0.300	4.983	<0.001	0.097	0.225	
TLICS	−0.370	0.271	−0.077	−1.366	0.174	−0.904	0.165	
LSC	1.599	0.276	0.362	5.793	<0.001	1.054	2.144	
Age	0.128	0.023	0.325	5.516	<0.001	0.082	0.173	
Model 2								0.513
HC	0.135	0.033	0.251	4.072	<0.001	0.070	0.201	
TLICS	−0.309	0.269	−0.064	−1.148	0.253	−0.839	0.222	
LSC	1.549	0.277	0.351	5.589	<0.001	1.002	2.097	
BMD	−1.001	0.178	−0.346	−5.619	<0.001	−1.353	−0.649	

BMD = bone mineral density, CL = confidence interval, HC = value of height correction, LSC = the load sharing classification score, SC = standardized coefficients, Sig. = significance, Std = standard, TLICS = The Thoracolumbar Injury Classification and Severity score, UC = unstandardized coefficients.

### 3.3. Factors affecting the loss of kyphosis correction

**Figure 4.**
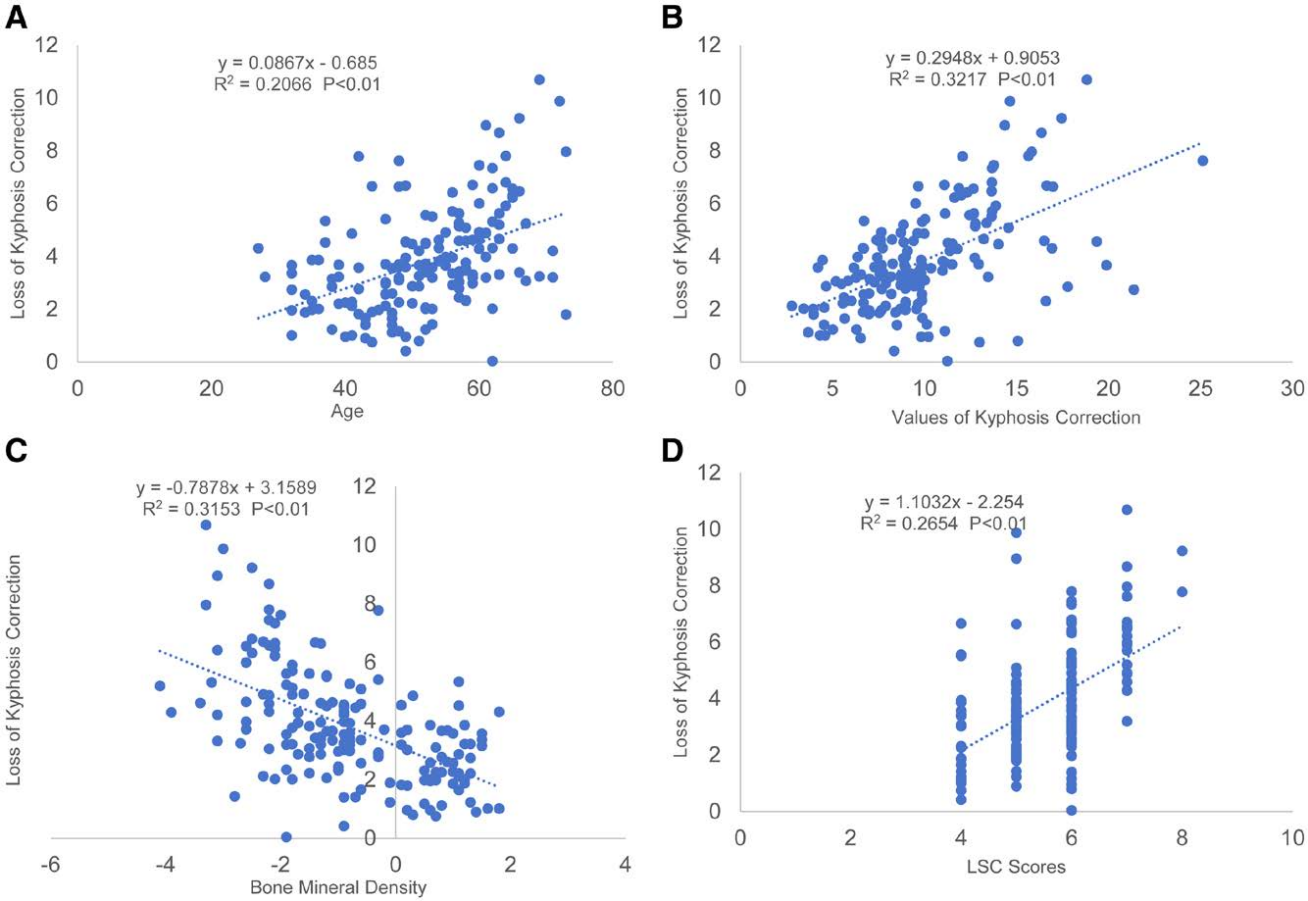
Scatter plots and linear regression values for the significant correlations with the loss of kyphosis correction. (A) A significant correlation is shown between the loss of kyphosis correction and the age of patients. (B) A significant correlation is shown between the loss of kyphosis correction and the values of kyphosis correction. (C) A significant correlation is shown between the loss of kyphosis correction and bone mineral density. (D) A significant correlation is shown between the loss of kyphosis correction and the LSC scores. LSC = the load sharing classification.

There was no significant sex difference in the loss of kyphosis correction (*P* > .05). Diabetes and decompressive laminectomy were risk factors in the loss of kyphosis correction (*P* < .05). Age, value of kyphosis correction and LSC scores were significantly and positively associated with loss of kyphosis correction, which was described by linear regression analysis to have a predictive value of 45.5% (Fig. 4A), 56.7% (Fig. 4B) and 51.5% (Fig. 4D) respectively. There was a significant and negative correlation between BMD and loss of kyphosis correction, with a 56.2% (Fig. 4C) predictive value. Neither TLICS scores (Fig. 3C) nor the time from fracture to surgery (Fig. 3D) was observed significant correlation to the loss of kyphosis correction. Simple linear regression analysis showed that age, value of height correction, LSC scores and BMD were all significant predictors of the loss of vertebral height correction.

Significant relationships (*P* < .05) were subjected to multivariate regression analysis. The results suggested age, BMD, value of height correction and LSC scores were constant significant predictors of the loss of kyphosis correction (Table 3).

**Table 3:** Multivariable linear regression to factors affecting the loss of kyphosis correction.

Parameters	UC		SC	t	Sig.	95% CL for Beta		Adjusted *R*^2^
	Beta	Std. Error	Beta			Lower bound	Upper bound	
Model 1								0.512
KC	0.232	0.031	0.447	7.491	<0.001	0.171	0.293	
LSC	0.468	0.135	0.219	3.463	0.001	0.201	0.735	
Age	0.065	0.011	0.340	5.866	<0.001	0.043	0.087	
Model 2								0.545
KC	0.211	0.030	0.407	7.062	<0.001	0.152	0.270	
LSC	0.405	0.132	0.189	3.083	0.002	0.146	0.665	
BMD	−0.563	0.080	−0.401	−6.997	<0.001	−0.722	−0.404	

BMD = bone mineral density, CL = confidence interval, KC = value of kyphosis correction, LSC = the load sharing classification score, SC = standardized coefficients, Sig. = significance, Std = standard, TLICS = The Thoracolumbar Injury Classification and Severity score, UC = unstandardized coefficients.

## 4. Discussion

In summary, there was no significant sex difference in loss of height or kyphosis correction. Diabetes and decompressive laminectomy were risk factors in the loss of correction. Age, LSC scores and values of height/kyphosis correction were significantly and positively correlated with the loss of vertebral height/kyphosis correction, while BMD had a significant and negative effect on it, respectively.

### 4.1. Status of posterior pedicle screw-rod fixation for thoracolumbar fracture

Thoracolumbar fractures are the most common spinal fractures, and the treatment of these fractures remains controversial.^[1,2]^ The main bone of contention focuses on conservative treatment or surgical treatment and the choice of operation methods.^[5]^ Conservative management including bracing, physiotherapy and manual therapy, and pharmacotherapy, has been proven effective in stable fractures by several studies.^[4,16]^ However, surgical treatment is more effective in correcting deformities and relieving pain, early mobilization and spinal canal decompression.^[8,17,18]^ Currently, posterior fixation with pedicle screws and rods can be used to manage most thoracolumbar fractures. However, the controversy over posterior short-segment or long-segment pedicle screw fixation continues. Dick et al first proposed the placement of intermediate screws at the fracture level in 1994,^[19]^ which had the advantages of increasing structural stiffness and protecting fractured vertebrae from anterior loading.^[3]^

With the popularity of minimally invasive concepts, percutaneous pedicle screw fixation with the advantages of shorter surgical time and hospitalization time, less intraoperative blood loss and infection rate, and less postoperative pain has been a common surgical option.^[20,21]^ However, the weakness of limited kyphosis and height correction should not be neglected.^[20]^ Its clinical effect needs to be further studied.

### 4.2. Risk factors for loss of correction

The addition of intermediate screws has been proven to increase the stability of constructs,^[9,10]^ and result in less postoperative pain, less implantation failure, and significantly better radiological results than short segment posterior fixation significantly.^[3]^ Norton RP et al^[22]^ found that the use of pedicle screws at the fracture level could optimize the internal fixation load and reduce the chance of collapsing kyphosis by biomechanical analysis. However, there was still a significant loss of vertebral height and kyphosis correction, which ranged from 3.7% to 6.1%^[20,23–25]^ in height and 0.9° to 5.4° in kyphosis.^[9,20,23–26]^ In this research, the values were 2.3% and 3.6°, respectively. The results suggested that age, BMD, value of correction and LSC scores were significant predictors, however, the TLICS score seems to have a weak role as a predictor of loss after surgery. LSC emphasizes fracture morphology which plays a major role in the loss of height and kyphosis correction. Wang et al demonstrated that there was a significant positive correlation between the LSC score and spinal instability.^[27]^ The TLICS emphasizes the initial neurological status and structural integrity of the posterior ligament complex over fracture morphology,^[21]^ and thus plays a minor role. Patients with old age, low BMD and high LSC scores are at high risk of loss of height and kyphosis correction. For these patients, appropriate clinical measures such as appropriate bed rest, delayed weight bearing, and long segment fixation must be taken to reduce the loss of correction.

In this study, the LSC score was the most effective predictor of loss of vertebral height and kyphosis correction. The association between the LSC score and loss of correction has been reported in several previous studies. Kose KC et al^[28]^ retrospectively evaluated 39 patients with type-A fractures and concluded that there was no difference between the low load sharing score group (LSC score ≤ 6) and high scores group (LSC score ≥ 7). However, Ferran Pellisé et al^[29]^ reported that the loss of surgical correction was significantly associated with the load-sharing score in 2014. Kim GW et al^[23]^ described that kyphosis recurrence was significantly associated with the LSC score but not with the TLICS score. Similar results were reported in our study.

### 4.3. To reduce the occurrence of correction loss

In order to reduce the occurrence of correction loss, several attempts have been made. Fu-Cheng Kao et al^[30]^ described that the use of bone cement intranspedicle intervertebral fixation could increase the stability of thoracolumbar burst fractures, partially restore the vertebral height, and decrease the curvature and movement of pedicle screws. This ultimately helped to prevent loss of correction in the long term. Another study^[31]^ demonstrated that transpedicle balloon-assisted reduction in combination with anterior column calcium phosphate bone cement could maintain a good reduction in unstable thoracolumbar burst fractures with a low incidence of loss of correction. Zhi-Wen Luo et al found that loss of correction could be decreased by transpedicular bone grafting.^[32]^ Besides, previous studies had proved that long-segment fixation was more effective than short-segment intermediate screw fixation in correcting local kyphotic Cobb angle and was more effective in reducing correction loss.^[33]^

### 4.4. Limitations

There are several limitations to this research. First, the included patient population was small. Second, the retrospective studies have inherent methodological limitations. In addition, the follow-up time (6 months) was short. More clinical factors such as the effects of disc rupture and endplate fracture needed to be included in future studies. How to reduce the loss of height and kyphosis correction also needed to be solved by further research.

## 5. Conclusion

Patients with older age, lower BMD, higher LSC scores, laminectomy and diabetes are at higher risk of vertebral height and kyphosis correction loss increase. For these patients, appropriate clinical measures such as long segment fixation, control of blood glucose, and increase of bone density must be taken to reduce the loss of correction. In addition, more clinical factors such as the time from fracture to surgery and the effect of medication need to be included in future studies.

## Author contributions

**Conceptualization:** Yi Mao.

**Data curation:** Junchao Zhang, Zhou Ye, Yi Mao.

**Formal analysis:** Junchao Zhang.

**Investigation:** Zhou Ye.

**Supervision:** Yi Mao.

**Validation:** Junchao Zhang, Yi Mao.

**Writing – original draft:** Junchao Zhang, Yi Mao.

**Writing – review & editing:** Zhou Ye, Yi Mao.
